# Telomeres in Space

**DOI:** 10.1111/acel.70030

**Published:** 2025-03-01

**Authors:** Abraham Aviv, Simon Verhulst

**Affiliations:** ^1^ Center of Human Development and Aging, New Jersey Medical School Rutgers State University of New Jersey Newark New Jersey USA; ^2^ Groningen Institute for Evolutionary Life Sciences University of Groningen Groningen the Netherlands

**Keywords:** astronauts, lymphocytes, neutrophils, space, telomeres

## Abstract

Recent studies have reported that the spaceflight environment lengthens leukocyte telomeres. We propose that this baffling finding reflects changes in the composition of leukocyte subsets rather than an actual increase in telomere length within individual leukocytes. Since leukocyte telomere length is associated with aging‐related diseases and longevity in humans, it is crucial to understand the underlying factors driving telomere length changes in space.

## Graphical abstract

Leukocyte telomeres shorten with age, but telomere length in adults is longer in neutrophils than in lymphocytes. Leukocyte telomeres are longer in space because of the neutrophil–lymphocyte ratio (NLR). A rise in the NLR explains the apparent telomere lengthening in space and its rapid shortening upon return to Earth.

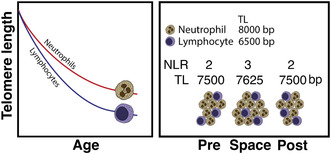

## 1 Introduction

The recently published Space Omics and Medical Atlas (SOMA) provides a valuable resource on the biological responses of humans to the space environment (Overbey et al. 2024). Among its findings are new data suggesting that the average telomere length (TL) in leukocytes increased during the Inspiration4 mission, a space flight lasting just 3 days (Garcia‐Medina et al. 2024). Similarly, half a decade ago, the NASA Twins Study reported an increase in the average leukocyte TL after a 1‐year space mission (Garrett‐Bakelman et al. 2019). Based on these studies, the public and much of the scientific community may presume that leukocyte telomeres lengthen in space. We suggest an explanation for the apparent lengthening of the average leukocyte TL in space and its rapid shortening upon return to Earth.

Population studies show that the leukocyte TL is associated with aging‐related diseases, principally cancer and atherosclerotic cardiovascular disease, and that it may even play an active role in their development (Codd et al. 2021; Haycock et al. 2017). These two disease categories broadly define human longevity, which is associated with leukocyte TL in middle‐high‐income societies (Codd et al. 2021; Steenstrup et al. 2017). Therefore, factors affecting the average leukocyte TL are crucial to understand because of the important implications of this complex phenotype for human health and, perhaps, lifespan.

Humans have evolved through interactions with Earth's environments, and our current understanding of telomere biology is based on terrestrial principles (Perlman 2011). Consequently, human telomeres' short‐ and long‐term responses to space are unpredictable and counterintuitive. Nevertheless, we draw on knowledge of telomere biology on Earth to guide our exploration of human telomeres in space.

Accordingly, we propose that the observed lengthening of leukocyte telomeres during space missions primarily stems from altered compositions of leukocyte subsets in the blood rather than the actual lengthening of telomeres in individual leukocytes. To explain this core idea, we first review key features of telomeres in human leukocytes on Earth and contrast them with those reported in astronauts. We then present our explanation for the observed telomere lengthening during space missions, offer suggestions for TL measurements in future space missions, and provide a brief conclusion.

## 2 TL Parameters in Human Leukocytes on Earth and in Space

The average length of leukocyte telomeres is highly variable in humans, with a standard deviation of about 700 base pairs (bp) at any age, starting at birth (Aubert et al. 2012; Benetos et al. 2013; Factor‐Litvak et al. 2016). It shortens with age—rapidly and non‐linearly during growth (about 1.5 kilobases [kb] in the first two decades of life) (Aubert et al. 2012; Factor‐Litvak et al. 2016; Lai et al. 2023) and then more slowly (about 25–30 bp/year) throughout adulthood (Benetos et al. 2013). The shortening of leukocyte telomeres with age reflects the replication of hematopoietic cells both within and outside the bone marrow. This occurs because telomerase, the enzyme responsible for elongating telomeres (Blackburn 2005), is repressed, though not entirely abolished (Yashima et al. 1998), in somatic cells.

A longstanding question in population studies is whether environmental exposures affect leukocyte TL in adult humans. A body of data shows that such effects are small in middle‐to‐high‐income societies (Aviv 2022; Bountziouka et al. 2022; Pepper et al. 2018). Thus, individuals born with either short or long leukocyte telomeres typically retain their relative short or long leukocyte telomeres throughout life, even though these telomeres progressively shorten with age (Aviv 2022; Benetos et al. 2013).

However, studies measuring TL in ‘whole blood’ (Garcia‐Medina et al. 2024) and in peripheral blood mononuclear cells (PBMCs) (Garrett‐Bakelman et al. 2019; Luxton, McKenna, Lewis, et al. 2020; Luxton, McKenna, Taylor, et al. 2020) have reported that the average leukocyte TL lengthens in space and rapidly shortens upon return to Earth. This prompts the question of whether the longer average leukocyte TL in astronauts' blood, which includes all leukocyte subsets and in PBMCs (70%–90% lymphocytes), reflects a genuine lengthening of leukocyte telomeres in space.

## 3 Likely Explanation for the Longer Leukocyte Telomeres in Space

Blood cells belong to two lineages: myeloid and lymphoid. The myeloid lineage comprises erythrocytes, neutrophils, eosinophils, basophils, monocytes, and dendritic cells. The lymphoid lineage includes T cells, B cells, and natural killer cells. Erythrocytes outnumber leukocytes by a thousandfold. However, since mammalian erythrocytes lack nuclei and, therefore, telomeres, the understanding of TL dynamics in the human hematopoietic system is principally based on TL measurements in leukocytes (Aviv 2023).

**FIGURE 1:**
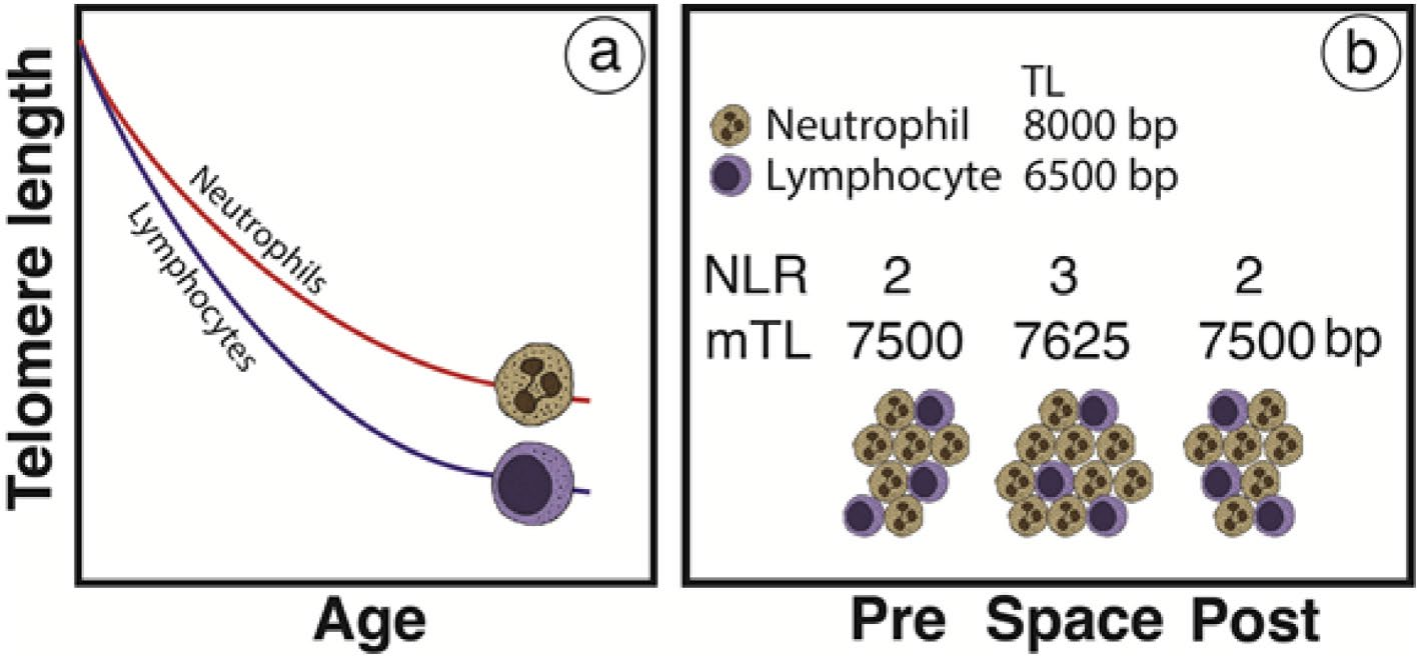
Characteristics of leukocyte telomere on Earth and in Space: (a) The shortening of telomeres with age in neutrophils (measured as TL in granulocytes), and lymphocytes; (b) An illustration showing the changes in the number of neutrophils, along with the resulting shifts in the neutrophil‐to‐lymphocyte ratio (NLR) and the mean leukocyte TL (mTL) between Earth and space. The average estimates for neutrophil TL and lymphocyte TL are based on a 50‐year‐old adult (Aubert et al. 2012).

Within an individual, TL varies across leukocyte subsets (Aubert et al. 2012), reflecting their replicative histories. Neutrophils, the predominant nucleated cells in the circulation, comprise about 60% of leukocytes. They are terminally differentiated and typically do not replicate after their release from the bone marrow (Kolaczkowska and Kubes 2013). In contrast, lymphocytes, constituting about 30% of leukocytes, continue to replicate and differentiate in extramedullary sites, including the thymus, in early life and other lymphoid organs throughout life. Therefore, telomeres in neutrophils or granulocytes (consisting of about 95% neutrophils) are longer than those in lymphocytes, and the difference in TL between these cell types increases with age, reaching a range of 1–2 kb (Figure 1a) (Aubert et al. 2012). This means that changes in the ratio of neutrophils to lymphocytes directly impact TL estimates that do not differentiate between leukocyte subsets. Moreover, TL varies among different types of lymphocytes; in human adults, it is longer in B cells than in T cells and in naïve T cells than in memory T cells (Aubert et al. 2012). The shorter telomeres in memory T cells than in naïve T cells reflect the formation of memory T cells through clonal expansion of naïve T cells (Kumar et al. 2018), a process that further shortens telomeres.

Environmental exposures that alter the composition of leukocyte subsets in the blood will impact the average leukocyte TL. An increase in the number of neutrophils will raise the neutrophil‐to‐lymphocyte ratio (NLR), leading to a longer average leukocyte TL. Conversely, decreasing the NLR will result in a shorter average leukocyte TL. As the NLR increases during space missions (Crucian et al. 2000, 2015; Paul et al. 2020; Stowe et al. 1999), changes in leukocyte composition might contribute to or fully explain the elongation of the average leukocyte TL in space and the rapid reversal of this effect after returning to Earth (Figure 1b). We note in this regard that on Earth, the NLR could rise with acute severe infection (Hajibandeh et al. 2020; Wu et al. 2024).

Highlighting the longer average leukocyte TL observed in blood samples obtained during the 3‐day Inspiration4 mission, the SOMA indicates that telomere lengthening occurs rapidly in response to spaceflight (Overbey et al. 2024). The authors of the Inspiration4 telomere study (Garcia‐Medina et al. 2024) suggest that the activation of the alternative lengthening of telomeres (ALT) pathway (Sobinoff and Pickett 2017) might explain this finding. Since telomere lengthening through ALT or telomerase typically occurs during mitosis, it is unlikely that these mechanisms could significantly lengthen telomeres in circulating leukocytes within just 3 days to a degree detectable by standard TL measurement methods. In addition, as leukocyte telomere shortening in adults is extremely slow on Earth (Benetos et al. 2013), what mechanisms could account for the rapid shortening of average leukocyte TL upon returning to Earth apart from changes in leukocyte composition after both long missions (Crucian et al. 2015; Paul et al. 2020) and short missions (lasting 8–18 days) (Crucian et al. 2000; Stowe et al. 1999)?

It is important to consider that not only changes in the NLR but also shifts in the proportions of lymphocyte subsets occur in space (Crucian et al. 2000, 2015). These include elevated levels of naïve T cells (Crucian et al. 2015), whose telomeres are much longer than those of memory T cells. These changes may also contribute to lengthening the average TL of leukocytes in space. Notably, Luxton, McKenna, Lewis, et al. (2020) proposed that “redistribution of leukocyte subsets,” in addition to other potential mechanisms (active ALT pathway, oxidative damage, senescence/apoptosis, radiosensitivity), might account for leukocyte telomere elongation in space. However, they did not substantiate this claim, leaving the meaning of “redistribution of leukocyte subsets” unclear.

The notion that leukocyte telomeres lengthen in space was primarily based on measurements of TL using quantitative polymerase chain reaction (qPCR) (Cawthon 2009). TL measurements with telomere fluorescence in situ hybridization (Telo‐FISH) in metaphase T cells grown in culture and DNA sequencing in CD4 cells further supported this idea (Garrett‐Bakelman et al. 2019; Luxton, McKenna, Lewis, et al. 2020). However, these measurements were performed in samples taken from one astronaut in space and his twin on Earth. Moreover, metaphase cells are selected cells capable of replicating in culture and are thus not representative of all circulating lymphocytes.

## 4 The Need to Know the Magnitude of the Effect of Space on TL

The measurements of leukocyte TL before, during, and after space missions were performed by qPCR, Telo‐FISH, and DNA sequencing. Measurements of TL by qPCR generate “unit free” TL data, expressed in telomere to single‐gene ratio metric. While Telo‐FISH and DNA sequencing generate data in absolute units of TL (bp or kb), the effects of space missions on TL measured by these methods were expressed in relative fluorescent intensity or “normalized” units. These amorphous metrics do not provide meaningful information about the potential health effects of space or other environments on telomeres. Without knowing TL results in absolute units, we cannot conclude the biological and medical implications of TL findings.

Finally, the 23 pairs of human chromosomes with q and p arms have 92 telomeres of varying lengths. Traditional measurement techniques typically provide the average TL across all chromosomes in a cell, yet the shortest among telomeres in the nucleus might limit cell replication (Hemann et al. 2001; Zou et al. 2004). Recent advancements in long‐read sequencing now enable the detection and measurement of telomeres specific to individual chromosomes (Karimian et al. 2024; Sanchez et al. 2024; Schmidt et al. 2024). As telomere research in humans expands into space, these innovative methods will be indispensable for assessing TL dynamics in astronauts' leukocytes, other somatic cells, and their germline cells, paving the way for groundbreaking insights both in space and on Earth.

## 5 Conclusion

Changes in the composition of leukocyte cell types provide the most parsimonious explanation for the puzzling findings of the rapid lengthening of the average leukocyte TL in space and its shortening upon return to Earth. Given the likely role of telomeres in various human diseases and longevity, it is crucial to understand the true impact of the space environment on TL dynamics in humans.

## Author Contributions

A.A. and S.V. have jointly written this perspective. They are accountable for all aspects of the paper and approved its final version.

## Conflicts of Interest

The authors declare no conflicts of interest.

## Data Availability

The authors have nothing to report.
